# Risk assessment and cost-effectiveness of animal health certification methods for livestock export in Somalia

**DOI:** 10.1016/j.prevetmed.2014.01.003

**Published:** 2014-03-01

**Authors:** T.J.D. Knight-Jones, F. Njeumi, A. Elsawalhy, J. Wabacha, J. Rushton

**Affiliations:** a The Pirbright Institute, Pirbright, United Kingdom; b The Royal Veterinary College (VEEPH), University of London, United Kingdom; c Food & Agriculture Organisation (AGAH), United Nations, Rome, Italy; d African Union – InterAfrican Bureau for Animal Resources, Nairobi, Kenya; e Faculty of Veterinary Medicine, Mansoura University, Mansoura, Egypt; f Faculty of Veterinary Medicine, University of Nairobi, Kenya

**Keywords:** Risk assessment, Cost-effectiveness, Somalia, RVF, FMD, Brucellosis, PPR, CCPP, CBPP

## Abstract

Livestock export is vital to the Somali economy. To protect Somali livestock exports from costly import bans used to control the international spread of disease, better certification of livestock health status is required. We performed quantitative risk assessment and cost-effectiveness analysis on different health certification protocols for Somali livestock exports for six transboundary diseases.

Examining stock at regional markets alone without port inspection and quarantine was inexpensive but was ineffective for all but contagious bovine pleuropneumonia, contagious caprine pleuropneumonia and peste des petits ruminants. While extended pre-export quarantine improves detection of infections that cause clinical disease, if biosecurity is suboptimal quarantine provides an opportunity for transmission and increased risk. Clinical examination, laboratory screening and vaccination of animals for key diseases before entry to the quarantine station reduced the risk of an exported animal being infected. If vaccination could be reliably performed weeks before arrival at quarantine its effect would be greatly enhanced.

The optimal certification method depends on the disease. Laboratory diagnostic testing was particularly important for detecting infections with limited clinical signs in male animals (only males are exported); for Rift Valley fever (RVF) the probability of detection was 99% or 0% with and without testing.

Based on our findings animal inspection and certification at regional markets combined with quarantine inspection and certification would reduce the risk of exporting infected animals and enhance disease control at the regional level. This is especially so for key priority diseases, that is RVF, foot-and-mouth disease and Brucellosis. Increased data collection and testing should be applied at point of production and export.

## 1 Introduction

The Somalia economy is highly dependent on livestock export to the Middle East, with over four million livestock (mostly sheep and goats) exported in 2010 (FSNAU, 2011). Importing nations have at times blocked this trade to prevent incursion of exotic pathogens (Davies, 2006; FEWS-NET, 2010; Abdo-Salem et al., 2011). Approximately 55% of Somalis are directly dependent upon livestock, with livestock exports accounting for 40% of the GDP (Knips, 2004; Qeiliye, 2008; Hamud, 2010). Loss of this export revenue due to prior trade bans has been disastrous for the Somali economy and the resulting reduction in supply has caused problems for importing countries (Davies, 2006; Abdo-Salem et al., 2011).

Currently, exported Somali livestock pass through privately owned quarantine stations, although an unknown number are exported informally without quarantine (Abdo-Salem et al., 2011) (Addis workshop, 2010 – see Section 2). Before animals gain entry to the quarantine they receive a pre-quarantine clinical inspection. Upon entry animals are treated for ectoparasites; diagnostic testing and vaccination are also carried out. Animals are examined throughout the quarantine period and rejected if diseased. The use of diagnostic tests, vaccination and length of quarantine vary according to the requirements of different importing nations. Animals found to be healthy are issued with a health certificate, allowing them to go for export to the specified country. Whether or not whole batches are rejected rather than individuals would depend upon the circumstances.

In some regions of Somalia animals may be clinically inspected at regional markets before reaching the quarantine. Healthy animals can then obtain a movement licence, allowing the animal or the batch to travel to the port.

There are important issues to keep in mind when considering sanitary controls for livestock export in Somalia. This livestock trade is of huge economic importance and with limited alternative economic opportunities it needs to be safeguarded. Blocking the trade not only increases poverty amongst the Somali people, but can actually increase the risk of disease spread through increases in smuggling and illegal livestock exports. Finally imposing export protocols that fully comply with OIE standards is challenging in Somalia.

In this paper we evaluate current export health certification methods based on port quarantine inspection and alternatives using upstream health certification at regional markets; minor variations in length of quarantine and laboratory testing protocols have also been assessed.

Recommendations made on adapted methods of livestock export disease control in a developing country like Somalia are specific to this compromised situation and, although they may relevant to other developing countries, should not be interpreted as more widely applicable.

## 2 Materials and methods

In the study a quantitative risk assessment combined with cost-effectiveness analysis has been used to evaluate the following different certification models:

• Certification based on procedures performed at the quarantine stations only.
• Certification based on procedures performed at the quarantine combined with regional market inspection.
• Certification based on regional market inspection only.

Export without certification has also been assessed for comparison; this could also be seen as the risk with no control measures as is the case with illegally exported animals. Length of quarantine period was 21 days unless stated otherwise.

The species and diseases considered were selected according to stakeholder's recommendations. Diseases included in the study are: foot-and-mouth disease [FMD] (cattle and sheep/goats), Brucellosis (cattle, sheep/goats and camels), contagious bovine pleuropneumonia [CBPP] (cattle), Rift Valley fever [RVF] (sheep/goats), contagious caprine pleuropneumonia [CCPP] (goats) and peste des petits ruminants [PPR] (sheep/goats).

For all the diseases and species considered the risk question was:

“What is the risk that an animal of species X, exported from Somalia, is infected with the causative agent of disease Y?”

Biological pathways were drawn up to describe the series of events required for an animal to be infected at the point of export. The estimated proportion of exported animals that were infected using a given certification method was compared to the proportion infected with no control measures in place. This risk difference was multiplied by the total number of exports from Bossaso and Berbera ports in Somalia in 2010, i.e. 3 919 218 shoats (sheep and goats), 227 611 cattle and 120 962 camels (FSNAU, 2011). This provided an estimate of the number of infected animals prevented from being exported per year by the various certification methods (compared to exporting with no control measures).

Quantitative data were used to provide values for the model input parameters; these were obtained from scientific publications, official reports, recorded data and expert opinion collected at two workshops (Addis workshop, 2010; see description below). When a design prevalence was required a low value was used reflecting the need to detect even low levels of disease and the expected low prevalence amongst largely clinically healthy export quality livestock.

Cost-effectiveness analysis was preferred to cost–benefit analysis as the future economic benefits were too complex and speculative to estimate. The measure of effectiveness in the cost-effectiveness analysis of the certification methods was taken as the reduction in the number of exported animals that were infected, any subsequent benefits are a function of this. Dividing the cost of each health certification method by the reduction in the number of infected animals being exported provided a measure of cost-effectiveness, i.e. the cost ($) per infected animal prevented from being exported (Knight-Jones et al., 2010); the smaller the Figure the more cost-effective (note: $ refers to US$ throughout the manuscript).

Cost was estimated as the price charged. For inspection performed at a regional market this was $15 per 100 cattle or camels and $5 per 100 shoats inspected; sheep and goats were treated as a single group (shoats). For quarantine there was some variation and distributions were used. The total quarantine fee for shoats, including inspection, testing, feed and housing, was $5–$8 per animal described by a Uniform(5,8) distribution and for cattle or camels quarantine fees were $18–$25 per animal described by a Uniform(18,25) distribution. These figures were obtained from experts at the Addis workshop (2010).

Relative cost-effectiveness was estimated as the cost-effectiveness for one control scenario divided by the cost-effectiveness for the baseline scenario.

Stochastic input parameters were incorporated using Monte Carlo simulation implemented in Excel (Microsoft Corporation) with @Risk (Palisade Corporation), using 10 000 iterations per simulation.

Sensitivity analysis was performed to see how variation in the input variables affected the results. This included probabilistic uncertainty analysis, where output values for each iteration were regressed onto the variable input parameters. Different quarantine lengths and laboratory testing protocols were also assessed. Only the most informative results of the sensitivity analysis have been reported.

### 2.1 Brucellosis

**Fig. 1:**
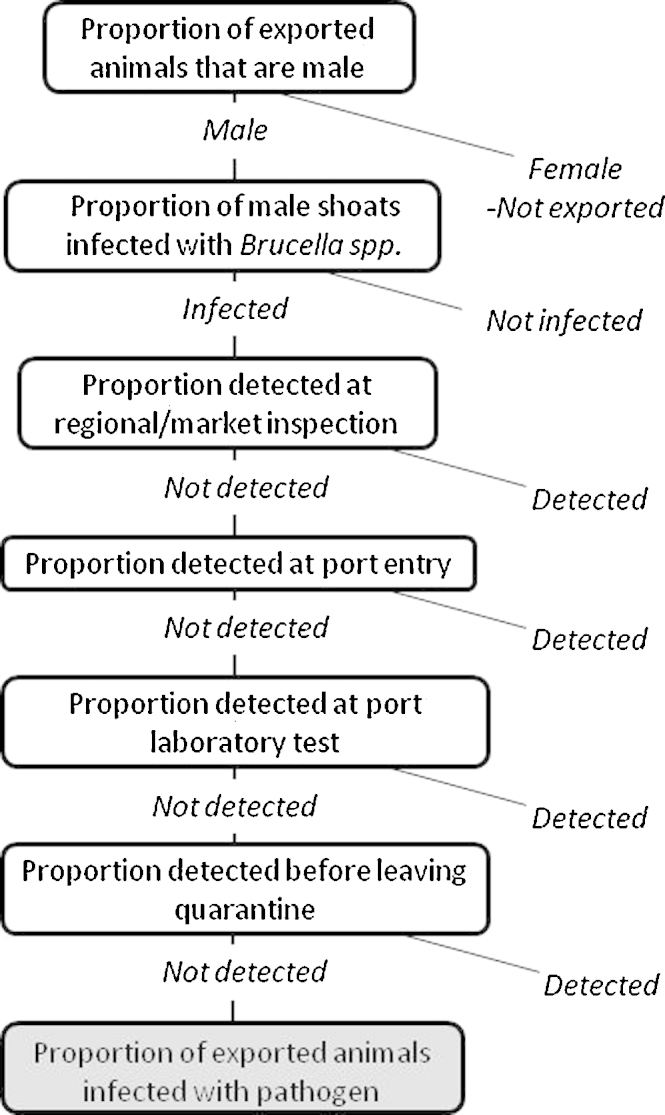
Risk pathway for the export of *Brucella* spp. infected livestock from Somalia.

The risk pathway for *Brucella* spp. is shown in Fig. 1. The model input parameters for shoats are shown in Appendix Table 1. All exported livestock are male. For the models with regional market inspection, all animals are assumed to be inspected. Once infected, animals are assumed to remain chronically infected and not recover during the quarantine period.

For cattle the proportion of males infected (*mi*) was estimated from an old survey of Somali livestock (Wernery et al., 1979), using a Uniform (0.021, 0.108) distribution. This reflects the prevalence for male bovines found in pastoralist herds and at a slaughter house, respectively. For camels the prevalence was estimated to be between 0.019 and 0.104 (Abbas and Agab, 2002); other input values were the same as for shoats.

Clinical examination, performed at the market, port entry and quarantine, were all assumed to have the same chance of detecting infection.

The model equations for the proportion of exports infected was the same for all species, as follows:

Quarantine procedures only=mi×me×mll×mq

Quarantine and regional/market procedures=mi×me×me×mll×mq

Regional/market procedures only=mi×me

No control measures=mi

where ***mi*** is the proportion initially infected, ***me*** is the proportion of infected not detected at a clinical exam, ***mll*** is the proportion of infected not detected by the laboratory test and ***mq*** is the proportion not detected by inspection during the quarantine period.

### 2.2 Foot-and-mouth disease

**Fig. 2:**
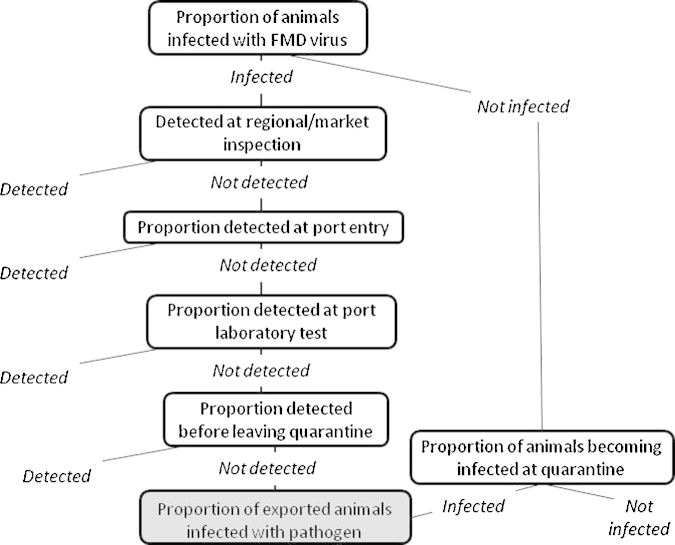
Risk pathway for the export of foot-and-mouth disease virus infected animals from Somalia; also used for Rift Valley fever, peste des petits ruminants, contagious caprine pleuropneumonia and contagious bovine pleuropneumonia.

The risk pathway for FMD is shown in Fig. 2. It differs from the pathway for Brucellosis as the latter needs to account for the fact that only males are considered for export and *Brucella* spp. risk will be lower in males. In addition the potential for within quarantine spread is far greater for FMD. The input parameters for FMD in shoats and cattle are shown in Appendix Tables 2 and 3, respectively. The model equations for the proportion infected were the same for cattle and shoats, as follows:

Quarantine procedures only– at quarantine entry=mi×me×mll

Quarantine and regional/market procedures– at quarantine entry=mi×me×me×mll

Regional/market procedures only=mi×me

No control measures=mi

For control scenarios involving quarantine, the above formulas only predict the proportion of infected animals that enter the quarantine. With highly infectious diseases like FMD there is a risk of transmission within the quarantine. Once an estimate of the proportion infected at the start of the quarantine was obtained an estimate of the proportion that would be infected at the end of quarantine was computed using a stochastic SIR state transition model. A quarantine batch size (*n*) of between 30 000 and 2 00 000 shoats [Uniform(30 000,200 000)] and 1000–10 000 cattle [Uniform(1000,10 000)] was used based on reported data (Addis workshop, 2010). The probability of receiving an effective contact from an infected individual per day (*β*) was given as *k*/(*n* − 1), where *k* was the number of effective contacts made by an individual within a herd per day. For cattle *k* = BetaPert(13.7, 21.8, 54.1) (Carpenter et al., 2004), for shoats this value was divided by four in line with other studies (Backer et al., 2009). The probability of receiving one or more effective contacts (*λ*(*t*)) was 1 − (1 − *β*)^*I*(*t*)^, where *I*(*t*) was the number of infected individuals on that day. The proportion of infected animals that recovered per day (*r*) was 1/*ds*, where *ds* is duration of infectiousness. Infected animals in quarantine are removed if detected, the proportion of infected animals detected each day was given as (1 − *me*) (this assumes all animals are inspected daily for signs of FMD).

The number of shoats in each state on a particular day (*t*) is then defined by the equations:

Susceptible:   S(t+1)=S(t)−λ(t)⋅S(t)

Infected:   I(t+1)=I(t)+λ(t)⋅S(t)−I(t)⋅r−([I(t)−I(t)⋅r]×(1−me))

Recovered:   R(t+1)=R(t)+I(t)⋅r+([I(t)−I(t)⋅r]×(1−me))

with *I*(*t*)·*r* being the number of animals recovered between *t* and *t* + 1 and [*I*(*t*) − *I*(*t*)·*r*] × (1 − *me*) being the number of infected animals detected between *t* and *t* + 1.

Transmission equations for cattle were identical to those used for shoats except vaccination was included, see equations below:

Susceptible:   S(t+1)=S(t)−λ(t)⋅S(t)−S(t)⋅vc⋅ve⋅vt(t)

where *vt*(*t*) was equal to 1 on the day of onset of vaccine immunity and was 0 on all other days. The day of onset of vaccine immunity was between 4 and 9 days after vaccination, with all days within this range equally likely.

Infected:   as   for   shoats.

Recovered:   R(t+1)=R(t)+I(t)⋅r+([I(t)−I(t)⋅r]x(1−me))+S(t)⋅vc⋅ve⋅vt

with *I*(*t*)·*r* being the number of animals recovered between *t* and *t* + 1 and [*I*(*t*) − *I*(*t*)·*r*] × (1 − *me*) being the number of infected animals detected between *t* and *t* + 1, and *S*(*t*)·*vc*·*ve*·*vt* being those becoming immune due to vaccination between *t* and *t* + 1.

No shoats but all cattle received laboratory tests at quarantine.

### 2.3 Rift Valley fever

The analysis focused on small ruminants as they cause the greatest public health concern. The same pathway described for FMD (Fig. 2) was used. Although not considered here, there is a risk of exportation of infected vectors associated with export of livestock from Somalia (Abdo-Salem et al., 2011). The model inputs for RVF are shown in Appendix Table 4.

RVF is spread via insect vectors (the most important route) or by direct contact (particularly via infected foetal membranes and fluids) (EFSA, 2005). Females are not present at the quarantines and insect vectors are not prevalent, in addition animals are treated with long acting insect repellents on arrival (Addis workshop, 2010), so the chance of transmission during quarantine was considered negligible and not incorporated.

The model equations were as for Brucellosis.

### 2.4 Peste des petits ruminants

The pathway described for FMD was used for PPR (Fig. 2). The model input parameters for PPR are shown in Appendix Table 5. Quarantine acquired infection is incorporated as a single time step (as opposed to the daily time steps required for FMD) as was detection during the quarantine. Recovery from infection was not considered due to prolonged shedding (Ezeibe et al., 2008).

The model equations for the proportion of exports infected were as follows:

Quarantine procedures only– at quarantine entry   (QR)=mi×me×mll

Quarantine procedures only– after quarantine=[QR+(1−QR)×qii]×mq

where ***qii*** is the proportion of animals not infected at quarantine entry that become infected during the quarantine period.

Quarantine and regional/market procedures– at quarantine entry   (QRm)=mi×me×me×mll

Quarantine and regional/market procedures– after quarantine=[QRm+(1−QRm)×qii]×mq

Regional/market procedures only=mi×me

No control measures applied=mi

Animals that die of the infection are included in those detected by clinical exam.

### 2.5 Contagious caprine pleuro-pneumonia

The pathway described for FMD was used for CCPP (Fig. 2). The input parameters for CCPP are shown in Appendix Table 6. As the primary host only goats were considered. In 2010 a total of 1 098 085 goats were exported (FSNAU, 2011). Although CCPP can occur in sheep they play an unclear and lesser role in disease transmission and have not been included.

The model equations for the proportion of exports infected were as follows:

Quarantine procedures only– at quarantine entry   (QR)=mi×me×mll

Quarantine and regional/market procedures– at quarantine entry   (QRm)=mi×me×me×mll

Regional/market procedures only=mi×me

No control measures applied=mi

The above two equations describe the proportion infected at entry to the quarantine. This was used as an input for an SIR state transition model to estimate the proportion infected after quarantine. With infectious diseases like CCPP there is a risk of transmission within the quarantine but recovery does not occur before exportation. Batch sizes (*n*) were as for FMD. Due to lack of data, the effective contact rate (ECR) was modelled as BetaPert(0.07, 0.126, 0.127) from a modelling study looking at CBPP (Mariner et al., 2006a,b). The proportion of susceptible infected per day *λ*(*t*) was then *ECR*·*I*(*t*)/*n*(*t*), where *I* is the number of infected animals. This is in line with the formulae used in the study from which the data were taken.

The number of animals in each state on a particular day (*t*) is then defined by the equations:

Susceptible:   S(t+1)=S(t)−λ(t)⋅S(t)

Infected:   I(t+1)=I(t)+λ(t)⋅S(t)

To adjust for the removal of detected initially infected animals during quarantine, the following equation was used:

I(final)=I(21)−[I(0)⋅mq]

Removed:   R(final)=I(0)⋅mq

where the final number infected [*I*(*final*)] is derived from the number infected at the end of quarantine [day 21 (*I*(21)], less those infected at the start of quarantine [*I*(21)], that were detected. The “Removed/detected” animals were dealt with in this way as new infections acquired during the quarantine would not be detected due to the long incubation period, however, animals already infected would be assessed throughout.

Quarantine was assessed with (1) no entry laboratory test and (2) with all animals tested at entry.

### 2.6 Contagious bovine pleuro-pneumonia

The pathway described for FMD was used for CBPP (Fig. 2). Formulae were as for CCPP, except using the quarantine batch size for cattle (*n*) of between 1000 and 10 000 (Uniform(1000,10 000)). The input parameters for the model are shown in Appendix Table 7.

### 2.7 Addis workshop

In order to obtain specific data and a clear understanding of livestock export in Somalia two workshops were held (Addis Ababa, Ethiopia, September and October 2010). The 18 participants came from Somaliland, Puntland and Southern and Central Somalia, and consisted of officials from the veterinary services, private veterinarians and staff from quarantine stations. Together they had significant experience of the different aspects of the trade, including market and port quarantine procedures.

Market chains were drawn up describing the production and export of livestock through Somalia. Participants were asked to provide uncertain estimates to quantify various aspects of livestock trade including information about diseases and their detection. Parameter estimates obtained from expert opinion at these workshops are referenced “(Addis workshop, 2010)”. Final distributions for parameter estimates were obtained by discussion and consensus using the six participants most involved in the practicalities of health certification in the different regions.

## 3 Results

### 3.1 Brucellosis

Market inspection was an ineffective way of detecting infection with *Brucella* spp. (Table 1); quarantine inspection with laboratory testing, although expensive, reduced the risk of exporting infected stock to a low level (90% range for infection risk at point of export = 0–4% approx.). If a high proportion of animals show clinical signs market inspection becomes more cost-effective compared to quarantine certification (standardised regression coefficient of 0.8 for relative cost-effectiveness for cattle). Uncertainty over the performance of the rose-bengal diagnostic test contributed to a lesser extent to uncertainty in relative cost-effectiveness; higher test sensitivity improved the cost-effectiveness of quarantine control.

### 3.2 FMD

Findings were similar for FMD (Table 2) where although certification with port quarantine procedures, including laboratory testing for cattle, costed approximately 20 times more per case detected (approximately $2000–$6000 per case detected), almost all cases were prevented from being exported, largely due to allowing sufficient time in quarantine for any infected animals to recover or be detected. With market inspection alone few cases were detected particularly for shoats which show limited clinical signs for FMD (most likely 0 cases detected, 90% range 0–8000 cases detected).

The potential for quarantine transmission was apparent. The model predicted that FMD outbreaks will occur in the quarantine and die out after 2–3 weeks of quarantine. This model assumes that all animals arrive at the quarantine at the same time; a continual supply of new susceptible animals would prevent the outbreak from burning out. Marginally reducing the number of animals infected at the start of quarantine through prior regional/market inspection has little to no impact on within quarantine FMD transmission. This is because any remaining infected animals rapidly infect other animals in the quarantine overwhelming any small risk reduction from prior market inspection. The pattern was similar for cattle and shoats. For cattle, without market inspection, quarantine outbreaks peaked at 49% infected at day 3 and fell to 19% at day 6 before burning out (results were similar for quarantine with market inspection).

The proportion of infected animals that showed clinical signs strongly influenced the risk of FMD in exported cattle (standardised regression coefficient of −0.9 for market inspection only). The uncertainty over the proportion of animals immune through prior infection at point of quarantine entry contributed to the uncertainty in risk estimates (standardised regression coefficient of 0.4). The timings of clinical disease and periods of viral shedding were also important with longer periods of infectiousness associated with greater risk when quarantine was used (standardised regression coefficient of 0.4 for shoats); when market inspection only was performed long infections (relating to longer periods of clinical disease) were associated with greater chance of detecting infected goats (standardised regression coefficient of −0.9 for market inspection only). This parameter was less uncertain for cattle and less influential.

### 3.3 RVF

Results for RVF are shown in Table 3. Combining market and quarantine had little effect on cost-effectiveness compared to quarantine inspection alone (relative cost-effectiveness = 0.99) as clinical examination did not detect RVF in males. The variation in the effect of quarantine on RVF risk was almost entirely due to the variation in the proportion that was laboratory tested with between zero and almost all 39 000 infected animals detected if no or all animals were tested, respectively. Some importing countries do not require RVF testing or only test a fraction of animals.

### 3.4 PPR

Results for PPR are shown in Table 4. If a combination of market inspection, port inspection and port laboratory testing is used with immediate shipment instead of observing a quarantine period, the risk of PPR virus infection is close to zero (90% range of 0–0.9%), resulting in 39 053 [most likely value] (90% range of 5854–38 955) infected shoats prevented from going for export. With normal 21 day quarantine length, within batch transmission could lead to increased risk of exporting infected animals, possible in large numbers, resulting in negative cost-effectiveness (i.e. compared to no control, for every additional infected shoat exported, on average $16 was spent on control). The inputs with the greatest effect on PPR risk for combined quarantine and market inspection were the proportion of infected that show clinical signs (*cs*) (standardised regression coefficient of −0.7), then the proportion of naïve that become infected during the quarantine (*qi*) (standardised regression coefficient of 0.58).

### 3.5 CCPP–CBPP

The threat of increased risk through within quarantine transmission also existed for CCPP and CBPP. With no laboratory testing within quarantine transmission could cause the proportion infected to double (CCPP) or triple (CBPP) (Table 5). For CCPP if the laboratory test was performed on all shoats in combination with market inspection and quarantine the percentage infected at export would be 0.2 (90% range of 0.1–0.6), resulting in 15 384 (90% range of 11 988–18 235) infected animals prevented from going for export (the lowest risk of all options). Uncertainty over the proportion of infected goats showing clinical CCPP explained much of the uncertainty in the output risk (standardised regression coefficient of −0.88 for market inspection only); this was less so when quarantine was used where variation in within quarantine contact rates had an appreciable effect (standardised regression coefficient of 0.5 for quarantine only).

For CBPP, if the laboratory test was performed on all cattle in combination with market inspection and quarantine the percentage infected at export would be 1.1% (90% range of 0.7–1.5%), resulting in 5357 (90% range of 4270–6172) infected prevented from going for export. Again this was probably the lowest risk option, although market inspection only also detected about 5000 infected animals for a fraction of the cost. If quarantine procedures were not applied variation in sensitivity of market inspection caused almost all the variation in CBPP risk. The degree of contact between animals determined much of the variation in risk when quarantine was used (standardised regression coefficient of 0.9, for quarantine without market inspection).

### 3.6 Cost-effectiveness analysis

Compared to quarantine procedures, market inspection alone is 5–10 times more cost-effective for *Brucella* spp. control, more than ten times more cost-effective for FMD in cattle and 16–17 times for FMD in shoats. That said, market inspection alone is not particularly effective at detecting infection with *Brucella* spp. or FMD virus, but it is relatively cheap. Market inspection is not expected to detect RVF cases, however, cost-effectiveness of quarantine with or without market inspection are similar ($619 versus $614 per infected export animal prevented, respectively) due to the relatively low cost of market inspection. For PPR quarantine had the potential to increase export infection risk giving negative cost-effectiveness estimates. Market inspection was highly cost-effective for PPR (most-likely $6 per infected animal prevented [90% range of $6–$117]) with moderate detection sensitivity (60%). Market inspection was also most effective and cost-effective for CCPP and CBPP costing on average $4 and $6 per case prevented, respectively. This compares to $561 and $1600 for CCPP and CBPP, respectively, if quarantine procedures were conducted in addition to market inspection.

## 4 Discussion

RVF poses the biggest threat to the international trade of Somali livestock (Abdo-Salem et al., 2011). As clinical examination is an ineffective way of detecting RVF in male animals, certification based on regional/market inspections does little to control the export of infected animals. Laboratory testing, although more costly, is the only effective means of detecting infected males. RVF in Somali livestock would be better controlled through early detection with effective surveillance and outbreak control (Davies, 2006).

FMD is widespread in Somalia, although there is huge variation in prevalence according to the serotype and region (Jabra, 2010). The role of live animal imports in the epidemiology of FMD in the Middle East has long been recognised (Hafez et al., 1994). FMD is a major trade issue in the region, with countries imposing consignment bans rather than wholesale movement bans. As clinical exam will not detect all infected animals, particularly in shoats and East African cattle breeds in regions where FMD is endemic, some infected animals are likely to make it into the quarantine station causing occasional outbreaks. Under OIE standard export procedures this would lead to rejection of the whole batch. When measures are less rigorous and only diseased individuals or small groups are rejected, some infected animals are likely remain undetected. Most but not all infected animals would recover during the quarantine period (in this example 21 days) and would no longer be viraemic when exported. Quarantine periods of 7 days are sometimes used which does not allow time for animals to clear the virus and for outbreaks to die out (Ithondeka, 2010). The issue of within quarantine transmission highlights the importance of biosecurity and quarantine batch rejection if risk is to be minimised; batch rejection also compensates for low detection rates at the individual animal level.

Yemen requires exported cattle to be vaccinated for FMD (Gazia, 2010). However, many animals may become infected by within quarantine transmission before the onset of immunity after vaccination (outbreaks would peak on day 3, whereas vaccine immunity would develop after at least 4 days). Vaccination a week, or ideally a month, before arrival at the quarantine would be hugely beneficial provided that effective FMD vaccines were used and could be kept at a suitable temperature (2–8 °C). Although FMD vaccination may limit clinical signs in infected animals, making them harder to detect, the reduction in virus shedding and transmission due to vaccination is more important in this context.

As an additional measure some countries test imported animals for serological evidence of historic FMD infection after arrival at the destination port. The merits of this could be questioned as Somalia and the importing countries have equivalent FMD status, however, serotype differences, only detectable with more extensive testing, may be important.

Clinical inspection appears to be effective for CCPP and CBPP. Importantly both can spread through close contact; they also have long incubation periods, meaning that new infections will not be detected during quarantine. As a consequence, market inspections with no quarantine results in the lowest risk and is the most cost-effective strategy. As quarantine may result in an increased risk of infection at export, the cost-effectiveness of this strategy is negative. A similar situation exists for PPR. This is contrary to the logic of standard pre-export quarantine where long quarantine periods are used to allow development of clinical signs and detection in those already infected.

One could argue that once the first case in a batch has been detected clinical examination will be performed more thoroughly and with higher probability of detection, particularly when performed repeatedly during a period of quarantine. Although this has not been considered explicitly in this study, variable probability of detection during inspection is included. Furthermore this probability is already quite high in the models used in this study (80–90% for all diseases except FMD which was 75–95%), leaving limited scope for improved inspection. The problem with clinical inspection is the proportion of subclinically infected animals that cannot be detected no matter how thorough the inspection is, especially with endemic diseases and vaccinated populations. Detection of infections that primarily cause clinical signs in pregnant females poses a challenge as only males are exported (e.g. RVF and Brucellosis).

For zoonotic pathogens such as *Brucella* spp. the opportunities for human infection from livestock exported through official channels should be limited as they are not intended for breeding, do not produce milk and often go directly to slaughter. The most likely exposure is to people involved in slaughter; however, it is of note that during the Hajj millions of pilgrims are involved in the slaughter of 10–15 million sheep and goats in Mecca (Davies, 2006). Transmission of RVF in this situation is a possibility and these risks may be increased by local insect vectors. Rapid slaughter of imported stock without contacting native stock is crucial to prevent transmission from infected imported animals to both humans and other animals.

The two main factors that determine the performance of the different methods of export disease control are:

(1) Can the INFECTION be effectively detected by clinical examination? and
(2) Will infection spread within the quarantine station?

Table 6 summarises which approach is optimal for controlling the different diseases based on these factors. For diseases with limited clinical signs regional market clinical inspection alone is ineffective.

The assessed diseases can be split into three categories based on characteristics of their risk of spread under quarantine conditions:

(1) Those where little or no transmission will occur in the quarantine (i.e. Brucellosis and RVF if vector transmission is controlled).
(2) Diseases where transmission can occur but recovery is quick (i.e. FMD, assuming persistently infected/carrier animals do not spread infection).
(3) Diseases where transmission can occur but recovery is not quick (i.e. PPR, CCPP and CBPP).

The results indicate that for category (1), quarantine reduces the risk. For category (2) a long period of quarantine would on the whole reduce the risk of infection (as any new infections have time to recover) but on occasion the risk may increase during quarantine, due to the unpredictable nature of these fast spreading pathogens. Even quarantine length is sufficient to allow recovery from viraemia, the percentage of animals that sero-convert will increase with quarantine duration and some countries reject animals based on serology. For category (3) the risk increases with increasing length of quarantine. Quarantine transmission can be reduced by using laboratory testing and vaccination of animals before admission to the quarantine.

In reality only one certification protocol can be applied per importing country. A possible way of selecting the most suitable export protocol would be to first prioritise the diseases based on the probability of importing infected Somali stock with no control measures and the consequences of pathogen incursion. Secondly, look at which protocols are effective at controlling the priority pathogens and thirdly consider cost-effectiveness if two protocols are similarly effective. Finally, the effect of the selected protocol on the control of lesser priority pathogens should be considered.

Zoonoses like RVF and Brucellosis typically cause considerable public concern. Looking at the non-zoonotic diseases considered, both PPR and FMD are endemic in the Middle East. However, unlike PPR, FMD has several serotypes, some exotic to the Middle East with the potential to cause heavy losses. CCPP and CBPP are both already endemic in the Middle East.

If the control of *Brucella* spp., RVF and FMD virus are prioritised then quarantine measures are required with laboratory diagnostics. Although market inspection is relatively cheap it is not an effective method of controlling these three pathogens. In the long term, if more control measures could be performed at regional markets, including diagnostics and vaccination before arrival at quarantine, it would reduce infection risk at point of export.

Improved disease control at regional markets would also benefit livestock health status throughout the country. If diseased stock can be detected at regional markets before they are transported across Somalia, then within country spread of disease will be reduced and with fewer infected animals entering the port, quarantine will be more effective.

Diseased consignments detected and rejected upon arrival at the destination port never return to Somalia but are shipped elsewhere in the region (Addis workshop, 2010). This highlights the importance of disease detection as far up the market chain as possible to minimise spread.

If a long quarantine compromises control of certain pathogens (e.g. PPR, CCPP and CBPP) the quarantine protocols should be amended to maintain control throughout the quarantine period. This could be evaluated through auditing and inspection, as well as diagnostic testing of imported Somali stock.

Some form of holding station has to exist to export animals in such large numbers. If quarantines were not used the alternative would be holding stations with minimal biosecurity and greater potential for spreading disease. In addition, it may not be possible to conduct inspection and certification to a consistent standard at regional markets throughout Somalia, thus a final evaluation of disease status is required before export. Therefore the use of quarantine at point of export is advocated despite its high cost.

Besides the measures investigated, discussions with Somali stakeholders indicated that animals are checked and screened at markets in order to select animals of export quality that are fit to travel. These practices are well known amongst the Somalis, but not documented and therefore not well understood by the importing countries. It is suggested that future local market level initiatives need to build on these local systems to collect data on the flows of animals, including the ones rejected and to perform sampling and vaccination.

When exporting many millions of animals from endemic populations in a poor country it may be unrealistic to expect them to be free from infection. In order to protect the Somali livestock export trade from further bans the number of infected, exported animals should be minimised by applying many different measures in combination. As the protocol that achieves maximal control for one disease may compromise the control of other diseases, prioritisation of pathogens is required to identify the preferred strategy.

## Figures and Tables

**Table A.1:** Input parameters for the risk assessment on *Brucella* sp. in exported Somali shoats.

Parameter (*symbol*)	Input	Source	Assumptions
Proportion of Somali shoats that are infected (*uc*)	Uniform(0.02,0.15)	McDermott and Arimi (2002)	Prevalence is similar to neighbouring countries as Somali surveillance data not available. Sero-positivity reflects active infection
Relative risk in males versus females (*rr*)	Expected value = 0.81	(Dinka and Chala, 2009) Where the natural log relative risk is normally distributed (*μ* = −0.21, *σ* = 0.18).	Based on data from cattle in Ethiopia. Truncated to avoid relative risks below zero
Proportion of males in populations used to estimate *uc* (*pm*)	Uniform(0.15,0.21)	Farm AFRICA (1996)	Based on data from neighbouring countries
Proportion of males infected (*mi*)	Expected value = 0.07	*uc* * *rr*/(1 − *pm*) + (*pm* × *rr*)	
Proportion of infected males with clinical signs (*cs*)	Uniform(0,0.1)	Addis workshop (2010)	Infected males show few clinical signs
Proportion of animals with clinical signs not detected at inspection (*mc*)	1 − Uniform(0.8,0.9)	Addis workshop (2010)	
Proportion of infected that are not detected at clinical exam (*me*)	Expected value = 0.96	(1 − *cs*) + (*cs* × *mc*)	
Proportion that are not detected by laboratory test, rose-bengal plate test (*mll*)	1 − Normal(0.812,0.248).	Gall and Nielsen (2004)	1 − test sensitivity. All animals are tested. Truncated to avoid sensitivities below zero
Proportion that are not detected during the quarantine period (*mq*)	Expected value = 0.95	(1 − *cs*) + (*cs* × *mc*^21^)	If quarantine is 21 days and inspections conducted daily

**Table A.2:** Input parameters for the risk assessment on foot-and-mouth disease in exported Somali shoats.

Parameter (*symbol*)	Input	Source	Assumptions
Proportion of shoats infected (*mi*)	0.01 (assumed design prevalence)	Cattle seroprevalence varies between 0% and 91.1%, depending on region and serotype (Jabra, 2010)	Seropositivity does not imply infection, so an assumed design prevalence of 0.01 (1%) was used to assess control measures
Time to appearance of clinical signs, days (*ip*)	Uniform(3,8)	Kitching and Hughes (2002)	Assumes values for Somali shoats are the same as non-pastoral FMD naïve sheep breeds
Number of days of viral shedding before clinical signs appear (*s*)	Uniform(1,2)	Sellers and Parker (1969)	
Start of viral shedding, days (*ss*)	Expected value = 4	*ip* − *s*	
Duration of viral shedding, days (*ds*)	Uniform(1,5)	Kitching and Hughes (2002)	
Proportion of infections that never show clinical signs (*sc*)	0.45	Kitching and Hughes (2002)	*25% no signs and 20% single mouth lesions. In endemic populations fewer may show clinical signs*
Proportion of the animals that develop clinical signs that are clinically affected on day of examination (*dc*)	Expected value = 0.2	(*ds* − *s*)/(*ss* + *ds*)	*Based on the fraction of the time an animal is infected that the animal has clinical signs*
Proportion of clinically detectable animals that are missed (*mc*)	1 − Uniform(0.75,0.95)	Addis workshop (2010)	Animals require full oral exam to reduce this (this is not performed)
Proportion of infected that are not detected at clinical exam (*me*)	Expected value = 0.9	((1 − *sc*) × *dc* × *mc*) + ((1 − *sc*) × (1 − *dc*)) + *sc*	
Proportion missed after lab test (*mll*)	1 (no sheep tested)	Gazia (2010)	No FMD laboratory testing is used for sheep
Length of quarantine (*nq*)	21 days	Recommended period (Thomson et al., 2009)	This in fact varies according to destination
Proportion immune in the population at large (*R*_*t=0*_)	Uniform(0,0.911)	Jabra (2010)	Seropositivity implies immunity. Seroprevalence in cattle used to represent shoats

**Table A.3:** Input parameters for the risk assessment on foot-and-mouth disease in exported Somali cattle.

Parameter (*symbol*)	Input	Source	Assumptions
Proportion of cattle infected (*mi*)	0.01	Seroprevalence varies between 0% and 91.1%, depending on region and serotype (Jabra, 2010)	Seropositivity does not imply infection, so an assumed value of 0.01 (1%) was used to assess control measures
Appearance of clinical signs, days (*ip*)	Betapert(2,6,14)	Alexandersen et al. (2003b)	
Start of viral shedding, days (*ss*)	*Ip*	Alexandersen et al. (2003b)	
Duration of viral shedding, days (*ds*)	Unform(4,5)	Alexandersen et al. (2003b)	
Proportion of infections that are subclinical (*sc*)	Triang(0.003,0.003,0.59183)	Hutber et al. (1999), Kitching (2002), and Alexandersen et al. (2003a)	Distribution Fitted from data in Hutber et al. (1999)
Proportion of animals that develop clinical signs that are clinically affected on day on examination (*dc*)	Expected value = 0.4	*ds*/(*ss* + *ds*)	Based on the fraction of the time an animal is infected that the animal has clinical signs
Proportion of clinically detectable animals that are missed (*mc*)	1 − Uniform(0.75,0.95)	Addis workshop (2010)	
Proportion of infected that are not detected at clinical exam (*me*)	Expected value = 93%	((1 − *sc*) × *dc* × *mc*) + ((1 − *sc*) × (1 − *dc*)) + *sc*	
Proportion receiving a laboratory test (*pl*)	1 ∼ all tested	Gazia (2010)	Assumed all tested for antibodies by ELISA
Proportion of tested, infected animals not detected by laboratory test (*ml*)	1 − Uniform(0.9,0.95)	Moonen et al. (2004) and Niedbalski (2004)	1 − test sensitivity
Proportion missed after lab test (*mll*)	Expected value = 0.075	(*pl* × *ml*) + (1 − *pl*)	
Proportion vaccinated (*vc*)	1	Gazia (2010)	All vaccinated – vaccine takes effect at day 4 of quarantine
Onset of vaccine immunity	Uniform(4,9)	Cox and Barnett (2009)	Immunity develops 4 to 9 days after vaccination
Vaccine efficacy (*ve*)	Uniform(0.5, 0.8)	Addis workshop (2010), Backer et al. (2009), and Cox and Barnett (2009)	Concerns protection against infection
Length of quarantine	21 days	Recommended period (Thomson et al., 2009)	This in fact varies according to destination
Proportion immune in the population at large (*R*_*t*=0_)	Uniform(0,0.911)	Jabra (2010)	Seropositivity implies immunity

**Table A.4:** Input parameters for the risk assessment on Rift Valley fever in exported Somali livestock.

Parameter (*symbol*)	Input	Source	Assumptions
Proportion of Somali shoats that are infected (*mi*)	0.01	Assumed design prevalence	RVF is believed to be endemic in Somalia but prevalence varies (Soumare et al., 2007)
Proportion of infected animals with clinical signs (*cs*)	0	Acha and Szyfres (2001), EFSA (2005), and Davies (2006)	Exported animals are males, which do not show clinical signs
Proportion of infected that are not detected at clinical exam (*me*)	1	1 − *cs*	
Proportion tested with ELISA antibody test at quarantine (*pl*)	Discrete({0,0.05,1},{1,1,1})	Gazia (2010)	Either 0%, 5% or 100% of animals are tested
Proportion of infected and tested that are not detected by laboratory test (*ml*)	1 − (RiskUniform(0.99,1))	Paweska et al. (2003) and Paweska et al. (2005)	1 − test sensitivity
Proportion missed after lab test (*mll*)	Expected value = 0.65	(*pl* × *ml*) + (1 − *pl*)	
Proportion that are not detected during the quarantine period (*mq*)	Expected value = 1		Exported animals are males, which do not show clinical signs

**Table A.5:** Input parameters for the risk assessment on peste des petits ruminants in exported Somali shoats.

Parameter (*symbol*)	Input	Source	Assumptions
Proportion of Somali shoats that are infected (*mi*)	0.01	Assumed design prevalence	
Proportion of infected animals with clinical signs (cs)	Uniform(0,1)	This varies greatly, sheep may be asymptomatic (Kulkarni et al., 1996; OIE, 2009b)	
Proportion of clinical cases not detected at clinical exam (*mc*)	1 − Uniform(0.8,0.9)	Addis workshop (2010)	
Proportion of infected that are not detected at clinical exam (*me*)	Expected value = 0.575	(*cs* × *mc*) + (1 − *cs*)	
Proportion tested with ELISA antibody test at quarantine (*pl*)	Discrete({0,0.05,1},{1,1,1})	Gazia (2010)	Either 0%, 5% or 100% of animals are tested
Proportion of infected that are not detected by laboratory test (*ml*)	1–0.922	Singh et al. (2004)	No uncertainty incorporated
Proportion missed after lab test (*mll*)	Expected = 0.95	(*pl* × *ml*) + (1 − *pl*)	
Proportion of uninfected that are immune (*pi*)	1–0.29	Medina (2007b)	Based on 29% seroprevalence in Somalia
Proportion of naïve that become infected during the quarantine (*qi*)	Uniform(0.1,0.9)	Up to 90% morbidity reported (OIE, 2009b)	
Proportion of animals not infected at quarantine entry that become infected during the quarantine (*qii*)		*pi* × *qi*	
Proportion of infections not detected during the quarantine period (*mq*)	Expected value = 0.575	As for *me*	Simplification as infections are acquired throughout the quarantine

**Table A.6:** Input parameters for the risk assessment on contagious caprine pleuropneumonia in exported Somali shoats.

Parameter (*symbol*)	Input	Source	Assumptions
Proportion of Somali shoats that are infected (*mi*)	0.0169	Medina (2007a)	This is based on an apparent seroprevalence of 1.3% adjusted for false negatives
Proportion of infected animals with clinical signs (*cs*)	Uniform(0.8,1)	Up to 100% morbidity in goats (Rurangirwa et al., 1981; OIE, 2009a)	
Proportion of clinical cases not detected at clinical exam (*mc*)	1 − Uniform(0.8,0.9)	Addis workshop (2010)	
Proportion of infected that are not detected at clinical exam (*me*)	Expected value = 0.235	(*cs* × *mc*) + (1 − *cs*)	
Proportion tested with ELISA antibody test at quarantine (*pl*)	0% and 100% tested assessed	Gazia (2010)	Laboratory test not routinely performed but assessed here for interest
Proportion of infected that are not detected by laboratory test (*ml*)	1 − Uniform(0.64,0.7)	Bellini et al. (1998) and Medina (2007a)	1 − test sensitivity
Proportion missed after lab test (*mll*)		(*pl* × *ml*) + (1 − *pl*)	
Proportion of infections not detected during the quarantine period (*mq*)	Expected value = 0.1	(1 − *cs*) + (*cs* × *mc*^21^)	If quarantine is 21 days and inspections conducted daily. New infections acquired during the quarantine could not be detected due to the long incubation period

**Table A.7:** Input parameters for the risk assessment on contagious bovine pleuropneumonia in exported Somali cattle.

Parameter (*symbol*)	Input	Source	Assumptions
Proportion of Somali cattle that are infected (*mi*)	0.0338	Somali Animal Health Services Project (2008)	This is based on an apparent seroprevalence of 2.6% adjusted for false negatives
Proportion of infected animals with clinical signs (*cs*)	0.8	EU (2001)	No variation incorporated
Proportion of clinical cases not detected at clinical exam (*mc*)	1 − Uniform(0.8,0.9)	Addis workshop (2010)	
Proportion of infected that are not detected at clinical exam (*me*)	Expected value = 0.32	(*cs* × *mc*) + (1 − *cs*)	
Proportion tested with ELISA antibody test at quarantine (*pl*)	0% and 100% tested assessed	Gazia (2010)	Laboratory test not routinely performed but assessed here for interest
Proportion of infected that are not detected by laboratory test (*ml*)	1 − Uniform(0.64,0.7)	Bellini et al. (1998) and Medina (2007a)	1 − test sensitivity
Proportion missed after lab test (*mll*)		(*pl* × *ml*) + (1 − *pl*)	
Proportion of infections not detected during the quarantine period (*mq*)	Expected value = 0.2	(1 − *cs*) + (*cs* × *mc*^21^)	If quarantine is 21 days and inspections conducted daily. New infections acquired at quarantine could not be detected due to the long incubation period

**Table 1:** Risk and cost-effectiveness for *Brucella* spp. infection in Somali livestock at point of export under different control scenarios. The most likely value is given with the 90% range in brackets (within which we believe the actual value is likely to lie).

Species	Scenario	Risk (%)	Relative risk	Reduction in No. of infected animals exported compared to no control	Cost-effectiveness ($ per animal detected)	Relative cost-effectiveness
Shoat	Quarantine only	0 (0–4.7)	0 (0–0.6)	93 313 (61 993–444 143)	66.4 (52.9–405.2)	6 (0.7–15.4)
	Quarantine and market inspection	0 (0–4.5)	0 (0–0.5)	142 793 (62 968–445 650)	78.8 (53.1–396.5)	5.7 (0.7–14.8)
	Market inspection only	7.2 (2.1–12.4)	Baseline group	1512 (889–31 228)	7.7 (6.3–220.2)	Baseline group
	No control	5 (2.2–13)	1 (1–1.1)	(Expected 278 500 infected with no control)	–	–

Cattle	Quarantine only	0 (0–4.2)	0 (0–0.6)	9347 (5845–21 417)	275 (223–843)	5.9 (0.8–17.6)
	Quarantine and market inspection	0 (0–4)	0 (0–0.5)	9843 (6027–21 433)	276 (224–816)	9.7 (0.7–16.9)
	Market inspection only	5.5 (3.9–9.7)	Baseline group	320 (64–1518)	31.3 (22.5–534.3)	Baseline group
	No control	7.6 (4–10.2)	1 (1–1.1)	(Expected 16 160 infected with no control)	–	–

Camel	Quarantine only	0 (0–3.9)	0 (0–0.6)	4297 (2019–10 987)	312.1 (232.7–1306)	9.6 (0.8–17.5)
	Quarantine and market inspection	0 (0–3.8)	0 (0–0.5)	3023 (2071–11 001)	327 (234–1285.5)	9.6 (0.8–16.8)
	Market inspection only	5.6 (2.2–9.6)	Baseline group	127 (26–778)	28.5 (23.3–708.9)	Baseline group
	No control	4.9 (2.3–10)	1 (1–1.1)	(Expected 7439 infected with no control)	–	–

**Table 2:** Risk and cost-effectiveness for foot-and-mouth disease in Somali livestock at point of export under different control scenarios. The most likely value is given with the 90% range in brackets (within which we believe the actual value is likely to lie). Median is also shown for reduction in number infected as the distributions are extremely skewed.

Species	Scenario	Risk (%)	Relative risk	Reduction in No. of infected animals exported compared to no control	Cost-effectiveness ($ per animal detected)	Relative cost-effectiveness
Shoats	Quarantine only	0 (0–0.1)	0 (0–0.1)	39 192 (35 301–39 192) Median = 38 853	620 (520–764)	16 (1.9–27.7)
	Quarantine and market inspection	0 (0–0.11)	0 (0–0.12)	39 192 (34 960–39 192) Median = 38 837	733 (526–769)	17 (1.8–28.3)
	Market inspection only	1 (0.8–1)	Baseline group	0 (0–8000) Median = 3891	33 (24–346)	Baseline group
	No control	1% design prevalence	1 (1–1.3)	(39 192 infected with no control)	–	–

Cattle	Quarantine only	0.03 (0.03–0.7)	0.04 (0.04–0.7)	2205 (719–2207) Median = 1908	2508 (1995–5948)	11 (1.4–27)
	Quarantine and market inspection	0.02 (0.03–0.7)	0.04 (0.04–0.7)	2226 (718–2207) Median = 1907	2526 (2011–5992)	11 (1.4–27)
	Market inspection only	0.99 (0.8–0.99)	Baseline group	57 (14–390) Median = 137	131 (88–2493)	Baseline group
	No control	1% design prevalence	1 (1–1.2)	(2276 infected with no control)	–	–

**Table 3:** Risk and cost-effectiveness for Rift Valley fever in Somali shoats at point of export under different control scenarios. The most likely value is given with the 90% range in brackets (within which we believe the actual value is likely to lie).

Scenario	Risk (%)	Relative risk	Reduction in No. of infected animals exported compared to no control	Cost-effectiveness ($ per animal detected)	Relative cost-effectiveness
Quarantine only	1 (0.001–1)^a^	1 (0.001–1)^a^	0 (0–39 134)^a^	614 (529–14 536)^a^	0.99 (0.99–0.99)
Quarantine and market inspection	1 (0.001–1)^a^	1 (0.001–1)^a^	0 (0–39 134)^a^	619 (533–14 636)^a^	Baseline group
Market inspection only	1 (1–1)	Baseline group	0	NA as zero effect	NA as zero effect
No control	1% design prevalence	1	(39 192 infected with no control)	–	–

a Highly bimodal resulting from if lab test was conducted or not.

**Table 4:** Risk and cost-effectiveness for peste des petits ruminants in Somali shoats at point of export under different control scenarios. The most likely value is given with the 90% range in brackets (within which we believe the actual value is likely to lie).

Scenario	Risk (%)	Relative risk	Reduction in No. of infected animals exported compared to no control	Cost-effectiveness ($ per animal detected)	Relative cost-effectiveness
Quarantine only	7 (4–48)	26 (10–61)	−231 777 (−1 841 505 to −120 315)	−16 (−200 to −13)	−0.05 (−29 to 0)
Quarantine and market inspection	6 (4–48)	59 (10–61)	−230 515 (−1 844 445 to −118 146)	−21 (−204 to −13)	−0.05 (−29 to 0)
Market inspection only	0.6 (0.2–0.95)	Baseline group	15 463 (1665–31 592)	6 (6–117)	Baseline group
No control	1	1 (1–5)	(39 192 infected with no control)	–	–

*Note*: Negative values imply greater risk than with no control measures.

**Table 5:** Risk and cost-effectiveness for contagious caprine pleuropneumonia and contagious bovine pleuropneumonia in Somali goats at point of export under different control scenarios (without laboratory testing). The most likely value is given with the 90% range in brackets (within which we believe the actual value is likely to lie).

Species	Scenario	Risk (%)	Relative risk	Reduction in No. of infected animals exported compared to no control	Cost-effectiveness ($ per animal detected)	Relative cost-effectiveness
CCPP	Quarantine only	3 (2–5)	10 (6 –11)	−20 490 (−40 001 to −3558)	−213 (−1423 to −159)	−51 (−405 to −37)
	Quarantine and market inspection	0.6 (0.3–2)	2 (1–3)	9384 (−220 to 15 366)	561 (−1764 to 3642)	152 (−393 to 862)
	Market inspection only	0.4 (0.2–0.5)	Baseline group	13 051 (12 064–16 631)	3.7 (3.5–4.4)	Baseline group
	No control	1.69	3 (3–7)	(18 558 infected with no control)	–	–

CBPP	Quarantine only	9 (7–11)	10 (6–10)	−14 606 (−18 471 to −7353)	−306 (−676 to −252)	−54 (−105 to −37)
	Quarantine and market inspection	3 (2–4)	3 (2–3.6)	934 (−2148 to 2894)	1600 (−22 796 to 22 926)	275 (−3418 to 3535)
	Market inspection only	1 (0.95–1.2)	Baseline group	4968 (4907–5569)	6.2 (6.2–6.9)	Baseline group
	No control	3.38	3 (3–3.5)	(7687 infected with no control)	–	–

*Note*: Negative values imply greater risk than with no control measures.

**Table 6:** Optimal livestock export control strategies (in italics) for different diseases categorised on both the effectiveness of clinical examination at detecting the disease and the risk of disease spread through transmission within a quarantine station.

	Negligible risk of spread at quarantine	High risk of spread at quarantine
Clinical diagnosis is partially effective	• *Clinical diagnosis*	• *Clinical diagnosis*
	• *Long quarantine*	• *Short quarantine*
	• *(Laboratory test beneficial)*	• *(Laboratory test beneficial)*
		e.g. *CCPP, CBPP and PPR*

Clinical diagnosis is not effective	• *Laboratory test essential*	• *Laboratory test essential*
	• *Clinical diagnosis*	• *Clinical diagnosis*
	• *Long quarantine*	• *Short quarantine*
	e.g. *RVF, Brucellosis*	e.g. *FMD (arguably long quarantine if rapid recovery)*

*Abbreviations*: Foot-and-mouth disease (FMD), Rift Valley fever (RVF), peste des petits ruminants (PPR), contagious caprine pleuropneumonia (CCPP), contagious bovine pleuropneumonia (CBPP).
